# Occult meningo-pial anastomosis, an under-recognized risk of non-target embolization in middle meningeal artery embolization for chronic subdural haematoma

**DOI:** 10.1186/s42155-025-00618-y

**Published:** 2025-10-28

**Authors:** Wan Lung Ryo Yeung, Kevan Juan Sham, Wai Lun Poon

**Affiliations:** 1 Department of Diagnostic and Interventional Radiology, Queen Elizabeth Hospital, 30 Gascoigne Road, Kowloon, Hong Kong SAR, China; 2 Department of Neurosurgery, Queen Elizabeth Hospital, Hong Kong, China

To the Editor,

Middle meningeal artery (MMA) embolization is an increasingly adopted adjunct or alternative treatment to conventional surgery for chronic subdural haematoma (cSDH). It has a favourable safety profile, with low recurrence rate [1]. While serious adverse events are uncommon [2], we report a rare case of an occult MMA-middle cerebral artery (MCA) anastomosis that was inadvertently embolized during MMA embolization.

An 80-year-old man presented with mild left-sided weakness 5 months after burr hole surgery for cSDH. Glascow Coma Scale (GCS) was 15 (maximum score). CT brain revealed cSDH recurrence with mass effect. The recurrence was likely precipitated by the initiation of dual antiplatelet therapy following recent coronary stent placement. Re-operation risk was considered high, and thus he was referred to us for MMA embolization.

Under local anaesthesia, the right external carotid artery (ECA) and MMA were cannulated with a 6Fr Neuron 053 catheter (Penumbra, Inc., United States) and a 1.7Fr Apollo™ microcatheter (Medtronic Neurovascular, United States). Right ECA and MMA angiograms showed no obvious orbital, petrosal or pial anastomosis. Embolization with Onyx™−18 (Medtronic Neurovascular, United States) was initiated in the right MMA parietal branch after the microcatheter was in wedged position. During injection, an unexpected curvilinear and medially oriented vascular channel was filled. Injection was then stopped immediately and the microcatheter was withdrawn under aspiration. A total volume of 0.9 mL Onyx-18 was injected. Post embolization fluoroscopy images and right common carotid artery (CCA) angiogram (Figs. 1 and 2) demonstrated Onyx extension into a right MCA distal parietal branch, with loss of contrast staining in part of the posterior right parietal lobe. The patient’s GCS remained full, and he was discharged home without new neurological deficit. Follow-up CT at 1 and 3 months (Fig. 3) showed hematoma reduction and a small right parietal infarct.

**Fig. 1:**
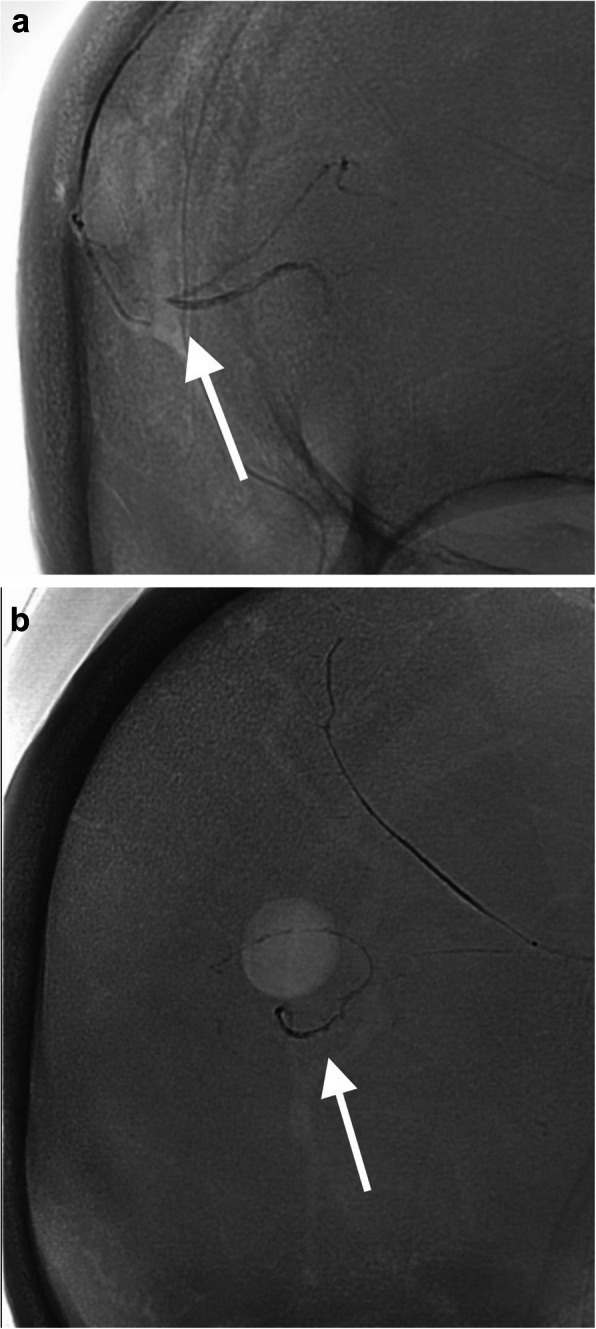
Magnified fluoroscopy images captured with biplane DSA machine (Philips Azurion 7 B20/15, Netherlands). A 1.7Fr Apollo™ microcatheter (Medtronic Neurovascular, United States) was parked at right MMA. **a** Magnified frontal projection and **b** lateral projection images showing a curvilinear, C-shaped and medially oriented opacity (white arrow) in proximity to the right parietal burr hole margin, indicative of Onyx cast extension into a right middle cerebral artery distal parietal cortical branch

**Fig. 2:**
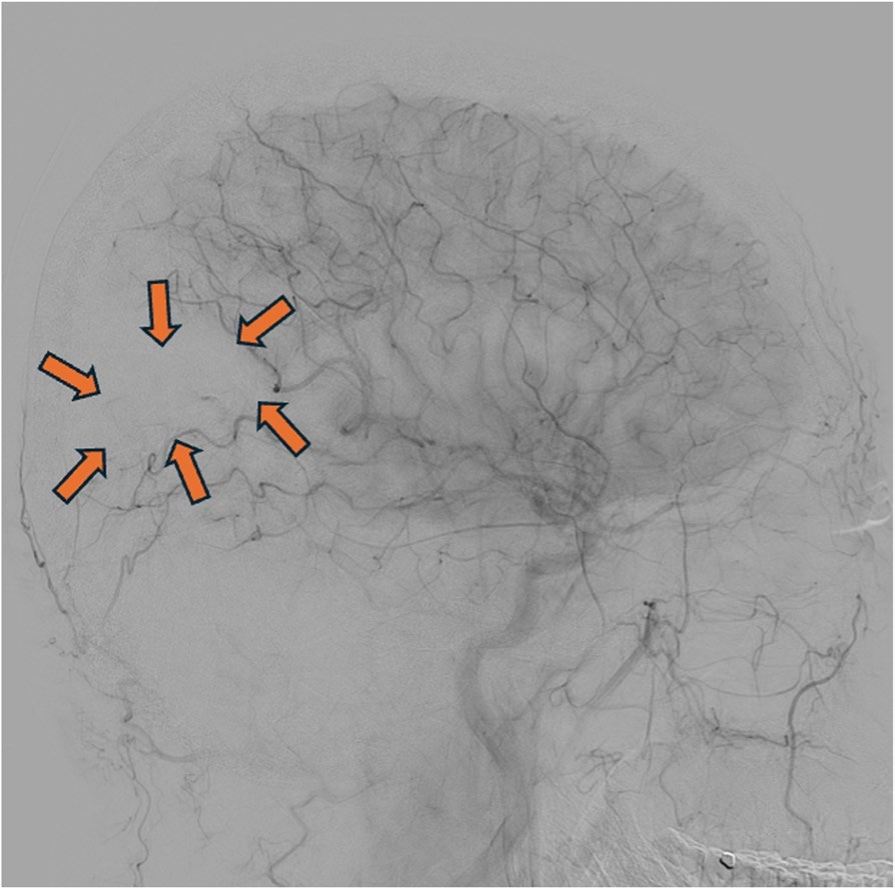
Digital subtraction angiogram (DSA) lateral projection image of right common carotid artery (CCA) run showing the absence of contrast staining in part of the right posterior parietal brain parenchyma (orange arrows)

**Fig. 3:**
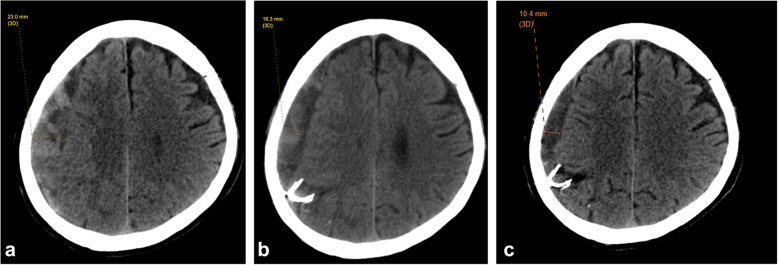
Serial CT brain selected images of the patient. **a** baseline **b** 1 month post MMA embolization and **c** 3 months post MMA embolization. There is gradual reduction in the thickness of the right chronic subdural haematoma on serial CT brain. Slender and C-shaped Onyx cast is seen at the posterior right parietal region with small area of infarct on 1 and 3 months post embolization CT brain images

Meningo-pial connections are commonly seen in dural arteriovenous fistula, chronic arterial occlusion and trauma, but can also develop after cranial surgeries. Burr hole surgery with dural exposure is used as an indirect revascularization strategy in Moyamoya disease because it can stimulate local angiogenesis and promotes ECA-pial anastomosis formation [3]. These collateral channels typically begin to develop within 2 weeks [4] and plateau in 3 to 6 months after surgery [5]. The effect of pial synangiosis is age-dependant [5], and thus the anastomosis in elderly patients tends to be smaller and less prominent than younger individuals [3]. In our case, both the patient’s advanced age and the prolonged interval between MMA embolization and surgery likely contributed to the formation of small meningo-pial anastomosis that had evaded detection on initial angiography. Besides, the microcatheter was positioned very distally in a wedged position (supplementary video). This raised the likelihood of opening collaterals and might have increased the risk of opening the meningo-pial anastomosis with subsequent embolization of the MCA cortical branches. Retrospective frame-by-frame inspection of the MMA angiographic run revealed subtle contrast wash-out near the burr hole margin (supplementary video), which suggests retrograde inflow from the right MCA distal parietal branch.

Careful patient selection, timing awareness and vigilant angiographic techniques are crucial to avoid non-target embolization. As shown in our patient, a prolonged interval between MMA embolization and burr hole surgery is a risk factor for the development of meningo-pial anastomosis. Therefore, the risks and benefits of embolization should always be weighed in this group of patients. In circumstances where delayed MMA embolization is planned, additional precautionary measures should be taken. These include 1) high frame rate super-selective MMA angiography with oblique projections to interrogate burr hole margins; 2) active search for subtle contrast wash-out or early venous opacification around burr hole margins; and 3) consideration of non-liquid embolic (coils or appropriately sized particles exceeding the suspected anastomotic caliber) when a meningo-pial communication is suspected.

Occult meningo-pial anastomosis is an under-recognized risk in delayed MMA embolization following burr hole surgery. Further studies are warranted to investigate the prevalence and temporal evolution of this anastomosis developed after cranial surgery.

## Supplementary Information

Supplementary Material 1: Video. Lateral projection of the super-selective right middle meningeal artery angiography (4 frames per second) run showing subtle contrast wash-out around right parietal burr hole margin, suggesting retrograde inflow from a distal parietal branch of right MCA.

## Data Availability

All data generated or analysed during this study are included in this published article and its supplementary information files.

## Abbreviations

MMA: Middle meningeal artery
cSDH: Chronic subdural hematoma
MCA: Middle cerebral artery
GCS: Glasgow Coma Scale
ECA: External carotid artery
CCA: Common carotid artery
